# Impact of the COVID-19 epidemic anxiety on college students' employment confidence and employment situation perception in China

**DOI:** 10.3389/fpsyg.2022.980634

**Published:** 2022-09-09

**Authors:** Sining Zheng, Guizhen Wu, Jiahao Zhao, Weiqi Chen

**Affiliations:** School of Public Management, Fujian Agriculture and Forestry University, Fuzhou, China

**Keywords:** COVID-19, epidemic anxiety, college student, employment, employment confidence, employment situation, employment guidance

## Abstract

The psychological problems and employment problems of college students have always been the focus of attention of all sectors of society. The COVID-19 epidemic has a great impact on the mental health and employment of Chinese college students. Under this background, this study discusses how epidemic anxiety affects the employment confidence and perception of employment situation of Chinese college students. Through the online questionnaire survey of 1,132 college students nationwide, and the ordinal logistic regression analysis of the survey data using Stata 16.0 software, the results show that: (1) Epidemic anxiety negatively affects Chinese college students' employment confidence and employment situation perception, and has a significant impact on employment confidence. The three control variables of employment guidance, older age and higher education have a significant positive impact on college students' employment confidence and employment situation perception. College students in the eastern region have stronger employment confidence and more optimistic employment situation perception. But the expected monthly salary is negatively correlated with employment confidence. (2) Male college students and Science and Engineering students' epidemic anxiety have a stronger negative impact on employment confidence and employment situation perception. (3) Employment guidance has a moderating effect on the relationship between epidemic anxiety, employment confidence and employment situation perception. Employment guidance can enhance college students' employment confidence and reduce their sense of employment crisis by alleviating epidemic anxiety. Combined with the research conclusions, it is proposed that the state and schools should pay attention to the psychological counseling of college students, strengthen the employment guidance of colleges and universities, vigorously support the development of small, medium-sized and micro enterprises, and improve the employment and entrepreneurship service system of college students, so as to promote the employment of college students.

## Introduction

The outbreak of COVID-19 pneumonia has had a strong impact on the global economic and social development. Up to now, COVID-19 is still sporadic throughout the country. Under the background of normalization of epidemic prevention and control, China's economy has gradually recovered, but the negative impact of the epidemic on college students' mental health and employment continues (Li et al., 2022). In 2022, the number of college graduates in China reached 10.76 million^1^, affected by multiple factors such as the COVID-19 epidemic and the economic downturn, the employment situation is severe and complex, and the uncertainty of job hunting for college graduates continues to increase (Wang and Li, 2022). On November 16, 2021, the Ministry of Education issued the notice on doing a good job in the employment and entrepreneurship of 2022 national college graduates^2^, aiming to improve the employment and entrepreneurship promotion mechanism, promote the quality and efficiency of employment and entrepreneurship, and promote the fuller and higher quality employment of college graduates.

The COVID-19 has affected large, medium and small enterprises in China to varying degrees, causing a large number of enterprises to face difficulties in survival and development (especially small, medium-sized, and micro enterprises). The phenomenon of layoffs, salary cuts and closures of enterprises occurs frequently, which directly leads to the contraction of market jobs. The surge in the number of returned overseas talents has also led to increased domestic employment competition and a worse employment environment (Li et al., 2020). In addition, domestic colleges and universities often take more strict epidemic prevention and control measures, which brings many inconveniences to college students' lives and job interviews. In this case, college students are prone to anxiety, worry and fear, which also affects the employment of college graduates (Wang Y. J. et al., 2021). Therefore, an in-depth study of college students' psychological problems and employment issues has important theoretical and practical significance.

This paper takes the COVID-19 as the background, takes Chinese college students as the research object, conducts an online questionnaire survey on college students in 36 colleges and universities across the country, and discusses the impact of COVID-19 anxiety on Chinese college students' employment confidence and employment situation perception through the combination of theory and data regression analysis. This study is expected to provide ideas or references for the state and colleges in carrying out college students' mental health education and improving college students' employment rate.

## Literature review

The COVID-19 has lasted for more than 2 years since its outbreak. The epidemic has not only caused heavy losses to the global economy and society, but also brought a huge negative impact on the mental health and employment of college students (Song et al., 2021). Looking at the existing research results, this paper combs the literature from the following three levels: (1) Research on the anxiety psychology of college students under the COVID-19. (2) Research on the impact of the COVID-19 on China's economy and employment. (3) Research on the employment of college graduates under the COVID-19.

From the research progress of the anxiety psychology of college students under the COVID-19, most studies have indicated that stress is the main factor causing anxiety (Aslam et al., 2021; Niu et al., 2021). As public health emergencies with strong infectivity and rapid spread, COVID-19 has caused a certain psychological burden on the public. As a special social group, As a special social group, college students' physical and mental development is not yet fully mature, and they are in a period of high incidence of psychological problems. Therefore, academic research on epidemic anxiety tend to focus on the mental health problems of college students during the epidemic (Wang J. et al., 2021). The research shows that the positive detection rates of anxiety and depression among college students are 10.2 and 19.33%, respectively. The COVID-19 epidemic has caused certain psychological disorders to college students at home, among which depression is more serious (Shader, 2020). The research indicates that the anxiety and depression levels of Chinese college students during the COVID-19 pandemic are higher than the national norm. The fear of the epidemic is a risk factor for anxiety. There are statistical differences between college students of different genders in the anxiety and panic of risk exposure. In the face of major stressful events, female's psychological coping ability is lower than male, moreover, female show more fear and tension than male and pay more attention to the epidemic situation, which may be related to female's more sensitive character (Liu et al., 2020). In addition, graduates are faced with issues such as graduation defense, employment, postgraduate entrance examination, and emotions, and they are under certain psychological pressure. However, due to COVID-19, various arrangements have been postponed repeatedly, which has affected graduation defense and employment of graduates. Therefore, graduates under COVID-19 are at higher risk of anxiety. In questioning how to alleviate anxiety during the epidemic, the study suggests that the online class mode, family relations and social epidemic prevention and control in colleges and universities have a significant impact on the level of epidemic anxiety (Tan et al., 2021). Schools should pay close attention to the physical and mental health of students, organize open classes of psychological counseling and convey positive information, and encourage college students to carry out more extracurricular activities to reduce the occurrence of mental health problems of college students during the epidemic (Liu, 2020; Rogowska et al., 2020).

From the research progress of the impact of the COVID-19 on China's economy and employment. The epidemic has had a devastating impact on human health and life. It has caused unprecedented disruptions in production, consumption, investment, supply chains, tourism, and trade, and it has had a profound effect on the economy of China and the world (Dewi and Melati, 2021; Ferreira et al., 2021; Karn, 2021). In terms of the impact of COVID-19 on labor demand and supply, market wage level and employment rate, some scholars found that COVID-19 had a dual impact on labor demand and supply. On the one hand, under the epidemic, the economic growth rate decreased, the demand for labor decreased, and the employment rate decreased. On the other hand, the decline of market wages has affected the labor participation rate and aggravated the reduction of the number of employed people (Zhang and Wu, 2020). Most studies have shown that although the impact of COVID-19 on employment is short-term, it is more serious than the impact of SARS in 2003 (Wang, 2020a). The short-term performance is employment reduction. The state should focus on migrant workers, college graduates, unemployed people and workers fighting the epidemic, and provide them with accurate and effective employment assistance services (Mo et al., 2020).

From the research progress of the employment of college graduates under the COVID-19. College graduates, as a special group in the employment, bear the double pressure of graduation and employment. COVID-19 undoubtedly further increases their psychological pressure on employment. The slowdown of labor mobility in the market, the decline of new jobs (Jin, 2020; Zhang and Zhao, 2020), and the increased uncertainty of employment have made college graduates worried, resulting in anxiety among college students (Cao, 2020). Generally, under the background of the COVID-19 outbreak, external environment factors, the psychological quality of college graduates, gap between high employment expectation and actual situation make employment difficult for college graduates. In addition, the epidemic has changed the career orientation of graduates. The security and stability of employment has become the first factor of employment. Graduates' confidence has also been affected to some extent. College graduates are anxious about employment prospects. Therefore, the government should establish a large employment database to accurately formulate employment policies (Ren, 2021). Colleges and universities should innovate employment work from the aspects of employment guidance ideas, employment network services, employment security, employment psychological counseling, employment assistance for special groups, play an intermediary role (Wang, 2020b), optimize the employment coping mechanism of graduates, improve the scientific cognition of the COVID-19, and promote the employment of graduates (Chen and Zhang, 2020).

To sum up, there are a lot of research results at home and abroad on the impact of COVID-19 on college students' mental health and employment, but most of them focus on one of the variables, such as COVID-19 and college students' psychological problems, or COVID-19 and employment relations. Few articles carry out in-depth research on the relationship between college students' psychological anxiety and employment psychological problems during the epidemic period, and the literature using large sample size data statistical analysis method is even rare. Therefore, this study supplements and expands the previous research topics and contents to explore the impact of epidemic anxiety on Chinese college students' employment confidence and employment situation perception.

## Theories and hypotheses

### Rational choice theory

Coleman first proposed the rational choice theory in 1990s. This theory mainly integrates the theories of micro and macro sociology, advocates using different components of the system, such as individuals, groups, organizations, institution, to explain the behavior of the system, which is called “internal analysis of system behavior” (Krstic and Krstic, 2015). The theory develops further based on the hypothesis of “economic man” in classical economics, and it extends the premise of economic man to “rational man” in neoclassical economics. The “rational man” hypothesis holds that in the process of participating in society, people will calculate and analyze rationally according to all aspects of information they have obtained, and make decisions that are most conducive to their own interests in order to obtain the maximum utility. Therefore, using this theory to construct the analytical framework of this paper can overcome the stereotype of economic man (Gao, 2012). Incorporating the research subject into the subjective perception of the epidemic environment can effectively analyze the obstacles faced by college students in the employment process under the epidemic environment. College students will rationally examine all types of resource, including material, spiritual, social needs and preferences, to find a job during the epidemic. If the available resources are uncontrollable, then the anxiety of employment will increase (Li et al., 2021).

### Theory of unsynchronization between economic and employment growth

Economic development is the result of the joint action of many production factors, such as capital, technology, land and labor. In the process of economic development, the higher the substitutability of other production factors to labor factors, the greater the contribution to economic development, and the lower the dependence of economic growth on labor. Therefore, economic and employment growth are not synchronized in real time. To solve this problem, researchers of this theory have focused on institutional factors and reduce the differences between individuals and society through institutions (Liu, 2010). After the institutional elements were put forward, China's economy and society have developed continuously and rapidly since the reform and opening up. It has continuously lowered the barriers of industrial structure transformation and upgrading from the institutional level, gradually transforming from the development of labor-intensive industries to the development of high-tech industries. It has also paid more attention to the investment and research on technological elements in economic development. Technological factors gradually replace labor factors to become the leading factor of economic development, and technological crowding out labor, making the employment situation of college students more and more severe (Zhang and Zhang, 2019). The sudden outbreak of the COVID-19 has led to a temporary slow development or even stagnation of China's economic development, forced many enterprises to speed up technological innovation, improve the core competitiveness of enterprises, reduce the impact of uncontrollable labor factors during the epidemic, avoid excessive dependence on labor, and expand the investment in technical factors again (Wu et al., 2022), which exacerbates the deterioration of the employment situation of college students and increases their employment anxiety.

### Career construction theory

Savickas, an occupational psychologist, observed that under the volatility, uncertainty, complexity, ambiguity (VUCA) scenario, people's career development produces obvious differentiation. Individuals who show strong adaptability in the face of major changes or crises related to their careers eventually achieve career success, whereas those who do not adapt well become victims of career crises. On this basis, Savickas proposed the theory of career construction, adaptation is defined and systematically classified, and the internal relations of adaptation are sorted out. It is proposed that occupational adaptation is a causal chain composed of adaptation preparation, adaptability, adaptive behavior and adaptive result. A high level of adaptation preparation, adaptability and adaptive behavior is conducive to obtaining the adaptive result (Savickas, 2013). The suddenness and unpredictability of COVID-19 fits the concept of VUCA scenarios. As the social, economic, and psychological changes caused by the pandemic will continue for some time, college students who have not yet been employed this year will still have to overcome the effects of the pandemic, whereas future graduates will continue to face uncertainties in the post-pandemic period (Pan et al., 2021). Therefore, the differences of college students' adaptation preparation, adaptability and adaptive behavior will directly affect the adaptive result and employment mentality.

**Figure 1:**
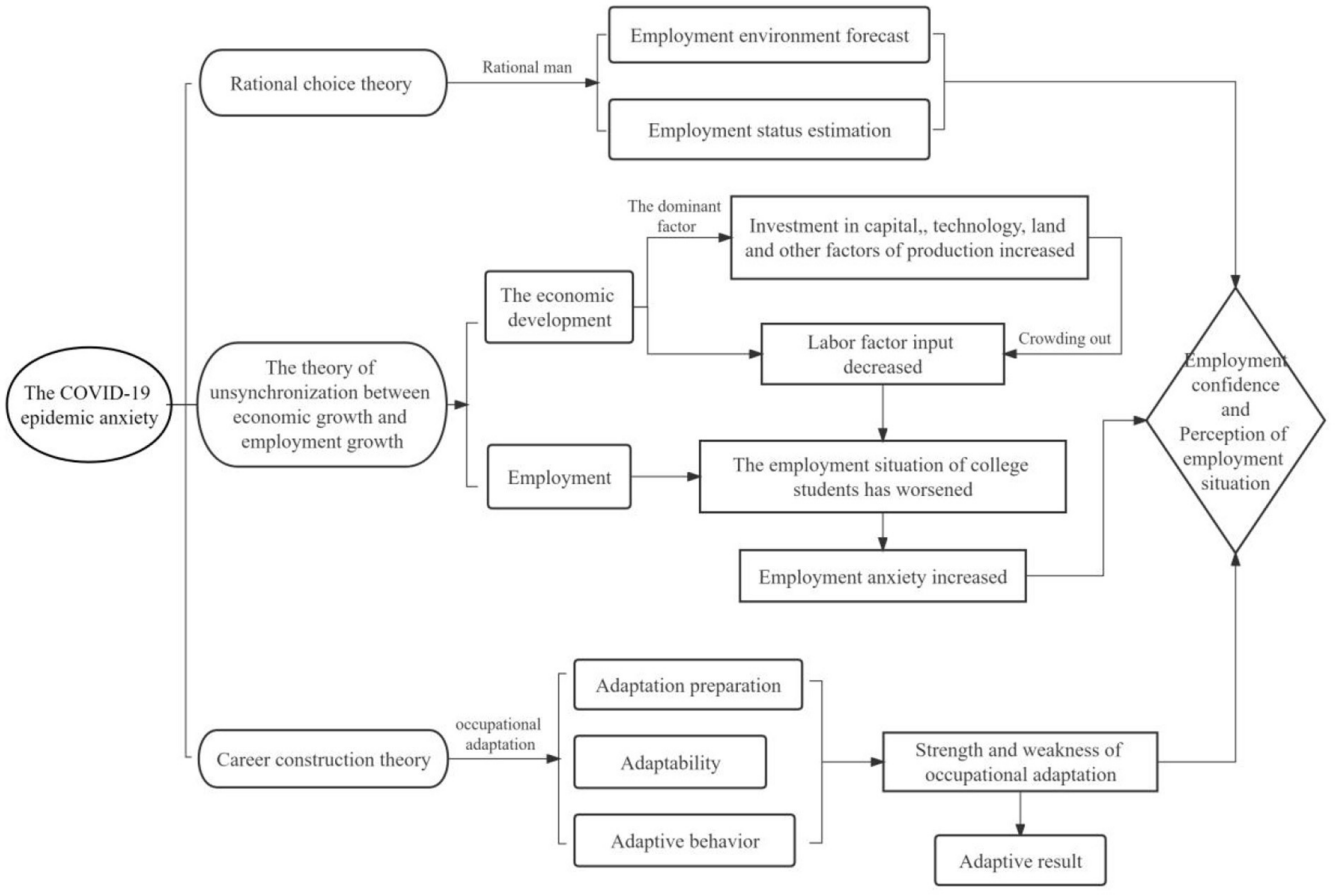
Theoretical framework diagram.

Based on the above analysis, this study constructs the theoretical framework of epidemic anxiety on college students' employment confidence and employment situation perception (Figure 1), and puts forward the following theoretical research assumptions (Table 1).

**Table 1:** Theoretical research assumptions.

Hypothesis 1	Epidemic anxiety negatively affects college students' employment confidence.
Hypothesis 2	The higher the epidemic anxiety, the stronger the sense of crisis of the employment situation of college graduates.
Hypothesis 3	Employment guidance can effectively help college students enhance employment confidence and reduce the sense of employment crisis.

## Methods

### Data collection and sample

The subjects are Chinese college students. Online questionnaires are distributed and collected through various mobile terminals (such as wechat, QQ, etc.), and participants fill in the questionnaire information online (platform: questionnaire star https://www.wjx.cn/). Random sampling, each IP address can only be filled in once, the survey time is set from September 2020 to October 2021, and 1132 valid samples are finally recovered. Participants are mostly concentrated in the eastern region of China, where the economy is relatively developed and many colleges and universities are gathered. A total of 36 colleges and universities are sampled, including 4 key colleges and universities, 6 sub key colleges and universities and 26 ordinary colleges and universities.

### Study content and measurements

The content of the questionnaire consists of three parts. The first part is the personal information of college students (not involving private information such as names), including “gender, age, major, region, employment status” and other information. The second part is the mental health status during the epidemic (evaluation contents: Kessler10 scale^3^), including “whether there is unexplained fatigue; whether there is often a sense of tension; whether it is difficult to calm down when there is tension; whether there is a sense of helplessness; whether there is a good rest; whether there is a sense of anxiety; whether there is often a sense of depression; whether there is a fear of difficulties in anything; whether there is no interest in anything; whether I feel worthless” a total of 10 evaluation contents. The third part is employment information, including “employment choices, employment plans, whether they have received employment guidance, perception of the employment situation during the epidemic and confidence in finding a satisfactory job”. The dependent variables, explanatory variables and control variables were measured according to the content of the questionnaire.

The measurement indicators of dependent variables are employment confidence and employment situation perception, which correspond to question 1 “do you think it is possible to find a satisfactory job under the epidemic?” and question 2 “how do you think the employment situation under the epidemic?”. Assign values to the options, and assign the options of employment confidence “very no confidence”, “relatively no confidence”, “general”, “relatively confident” and “very confident” to the ordered classification variables of 1–5, respectively. The higher the value, the stronger the confidence in employment and the confidence to find a satisfactory job. Similarly, the options of employment situation perception “very not optimistic”, “relatively not optimistic”, “general”, “relatively optimistic” and “very optimistic” are assigned to ordered classification variables of 1–5, respectively. The higher the value, the more optimistic the perception of employment situation and the weaker the sense of employment crisis.

The measurement index of the explanatory variable is epidemic anxiety, and the corresponding questions are the mental health evaluation contents of 10 items about anxiety and stress level in the second part of the questionnaire. The five options of each question are “Almost none”, “Occasionally”, “Sometimes”, “Most of the time” and “All the time”. The scores of each option are 1–5 points in turn, and the scores of the options of 10 questions are summed up. The higher the score, the stronger the anxiety level of college students. According to the total score, the epidemic anxiety level is further divided into four levels: Normal (10–15 points); Mild (16–21 points); Moderate (22–29 points); Serious (30–50 points).

The measurement indicators of control variables include employment guidance, age, education, major, expected monthly salary, region, etc. These variables may also affect college students' employment confidence and employment situation perception.

### Estimation

Because employment confidence and employment situation perception are ordered multi classification variables, this paper uses the ordinal multiple logistic regression model to estimate, and the model is constructed as follows:


(1) $$\begin{array}{r} employment_{\;}=\beta_{0\;}+\beta_{1\;}anxiety_{i}+X_{i}\delta +\mu_{i} \end{array}$$

Among them, “employment” represents the dependent variable: employment confidence or employment situation perception. Subscript “i” represents each college student sample. “anxiety” represents epidemic anxiety. “X” is the control variable. “μ” is the random error term. “β_0_” is the constant term. “β_1_” is the regression coefficient of the explanatory variable. And “δ” is the vector composed of the regression coefficient corresponding to the control variable.

### Statistical analysis

The data were statistically analyzed by Stata 16.0 software. First, descriptive statistical analysis of demographic characteristics and college students' mental health and employment status. Secondly, the ordinal multiple logistic regression method was used to analyze the impact of epidemic anxiety on Chinese college students' employment confidence and employment situation perception. Thirdly, according to the gender and major differences of Chinese college students, the explanatory variables and dependent variables are grouped and regressed to explore the impact of epidemic anxiety level of different groups on employment confidence and employment situation perception. Finally, through the following three-step regression method, the moderating effect of employment guidance on employment confidence and employment situation perception is analyzed: (1) College students are divided into two groups: those who have received employment guidance and those who have not received employment guidance. (2) Regression between employment guidance and epidemic anxiety separately, and analyze the impact of employment guidance on epidemic anxiety. (3) Interact epidemic anxiety with employment guidance to form an interactive item (epidemic anxiety ^*^ employment guidance), and then regression with employment confidence and employment situation perception, respectively.

## Results

demographic characteristics and selected characteristics of the study population are shown in Table 2. Among 1,132 college students, nearly 70% (67.93%) are from eastern China, and nearly two-thirds are female, of which 72.61% are unemployed. Most college students (74.03%) have mild or above epidemic anxiety, and their employment confidence is not too high. Most of them are in a general state (39.22%). 40.46% of college students believe that the current employment situation of the epidemic is not optimistic, and they have a strong sense of employment crisis. In terms of employment options, nearly half (48.67%) of college students choose to work in big cities, and the proportion of college students who expect a monthly salary of 5,000–8,000 yuan is relatively high (38.34%).

**Table 2:** Demographic characteristics of participants.

**Variable**	**Sample size** **(*N* = 1,132)**	**Proportion (%)**
**Epidemic anxiety**		
Normal (10–15 points)	294	25.97
Mild (16–21 points)	206	18.20
Moderate (22–29 points)	290	25.62
Serious (30–50 points)	342	30.21
**Gender**		
Female	716	63.25
Male	416	36.75
**Age**		
Under 18	17	1.50
18–25	862	76.15
26–30	197	17.40
Over 30	56	4.95
**Education**		
Junior college	595	52.56
Undergraduate college	417	36.84
Postgraduate	109	9.63
Doctoral candidate	11	0.97
**Major**		
Science and engineering	524	46.29
Liberal arts and history	608	53.71
**Employment status**		
Unemployed	822	72.61
Employed	310	27.39
**Employment confidence**		
Very no confidence	23	2.03
Relatively no confidence	82	7.24
General	444	39.22
Relatively confident	309	27.30
Very confident	274	24.20
**Employment situation perception**		
Very not optimistic	99	8.75
Relatively not optimistic	359	31.71
General	372	32.86
Relatively optimistic	160	14.13
Very optimistic	142	12.54
**Employment guidance**		
No	245	21.64
Yes	887	78.36
**Employment location preference**		
Small towns	96	8.48
Small and medium-sized cities	485	42.84
Big city	551	48.67
**Expected monthly salary (RMB)**		
Under 3,000	21	1.86
3,000–5,000	145	12.81
5,000–8,000	434	38.34
8,000–12,000	299	26.41
Over 12,000	233	20.58
**Region**		
Western	282	24.91
Central	81	7.16
Eastern	769	67.93

### Impact of epidemic anxiety on employment confidence and employment situation perception

Table 3 shows the relationship between epidemic anxiety and Chinese college students' employment confidence and employment situation perception. Epidemic anxiety significantly weakened college students' employment confidence (*p* < 0.01), hypothesis 1 was verified. At the same time, epidemic anxiety has a negative impact on college students' employment situation perception, but not significant, hypothesis 2 was verified. In addition, employment guidance and age increase significantly improved (*p* < 0.01) college students' employment confidence and employment situation perception. Higher education has a positive effect on employment confidence and employment situation perception, and has a significant promoting effect on employment confidence (*p* < 0.01). The higher the expected monthly salary, the lower the employment confidence. College students in eastern China have stronger confidence in employment and more optimistic employment situation perception.

**Table 3:** Impact of epidemic anxiety on employment confidence and employment situation perception.

**Variable name**		**Dependent variable**	
		**Employment confidence**	**Employment situation perception**
Explanatory variables	Epidemic anxiety	−0.407*** (−8.053)	−0.017 (−0.357)
Control variable	Employment guidance	0.977*** (6.990)	0.791*** (5.965)
	Age	1.112*** (9.505)	0.993*** (9.237)
	Education	0.331*** (3.836)	0.036 (0.446)
	Major	0.024 (0.216)	−0.229** (−2.092)
	Expected monthly salary	−0.042 (−0.748)	0.006 (0.114)
	Region	0.085 (1.298)	0.006 (0.098)
*N*	1,132	1,132

*p < 0.1, **p < 0.05, ***p < 0.01; Standard error of robustness in brackets.

### Impact of gender and major differences on employment confidence and employment situation perception

The relationship between gender and major differences and employment confidence and employment situation perception is shown in Table 4. Male college students' epidemic anxiety has a greater negative impact on employment confidence (*p* < 0.01) and employment situation perception (*p* < 0.1) than female college students. Compared with college students of Liberal Arts and History, the epidemic anxiety of college students of Science and Engineering is more likely to lead to lower confidence in employment (*p* < 0.01) and enhanced sense of crisis in employment situation (*p* < 0.05).

**Table 4:** Impact of gender and major differences on employment confidence and employment situation perception.

**Variable name**	**Employment confidence (gender)**		**Employment situation perception (gender)**		**Employment confidence (major)**		**Employment situation perception (major)**	
	**Female**	**Male**	**Female**	**Male**	**Science and** **Engineering**	**Liberal Arts** **and History**	**Science** **and Engineering**	**Liberal** **Arts and History**
Epidemic anxiety	−0.325*** (−4.974)	−0.564*** (−6.674)	−0.063 (1.001)	−0.126* (−1.655)	−0.429*** (−5.858)	−0.363*** (−5.140)	−0.156** (−2.241)	−0.143** (2.169)
Control variable	Controlled	Controlled	Controlled	Controlled	Controlled	Controlled	Controlled	Controlled
*N*	716	416	716	416	524	608	524	608

*p < 0.1, **p < 0.05, ***p < 0.01; Standard error of robustness in brackets.

### The moderating effect of employment guidance on employment confidence and employment situation perception

The results of three-step regression analysis in Table 5 show that employment guidance has a moderating effect on the relationship between epidemic anxiety, employment confidence and employment situation perception. First, the negative impact of epidemic anxiety on employment confidence and employment situation perception of college students who have received employment guidance is lower than that of college students who have not received employment guidance, Second, employment guidance can significantly reduce the epidemic anxiety of college students (*p* < 0.01), further alleviate their sense of employment crisis, and enhance their employment confidence. Third, the interaction between epidemic anxiety and employment guidance (epidemic anxiety ^*^ employment guidance) has a positive impact on college students' employment confidence and employment situation perception, hypothesis 3 was verified.

**Table 5:** The moderating effect of employment guidance on employment confidence and employment situation perception.

**Variable name**	**Step 1**				**Step 2**		**Step 3**
	**Employment confidence (employment guidance)**		**Employment situation perception (employment guidance)**		**Epidemic anxiety**	**Employment confidence**	**Employment situation perception**
	**Yes**	**No**	**Yes**	**No**			
Epidemic anxiety	−0.413*** (−7.328)	−0.436*** (−3.564)	−0.027 (−0.524)	−0.153 (1.299)		−0.486*** (−4.183)	−0.002 (−0.126)
Employment guidance					−0.436*** (−3.385)	0.711* (1.892)	0.539 (1.349)
Epidemic anxiety*employment guidance						0.097 (0.761)	0.011 (0.718)
Control variable	Controlled	Controlled	Controlled	Controlled	Controlled	Controlled	Controlled
*N*	887	245	887	245	1,132	1,132	1,132

*p < 0.1, **p < 0.05, ***p < 0.01; Standard error of robustness in brackets.

## Discussion

Research shows that the impact of COVID-19 on college students' mental health is multifaceted, mainly manifested in fear, anxiety, worry, etc. College students' mental health problems will also have a certain impact on their employment (Chen et al., 2020). The main purpose of this study is to analyze the impact of college students' epidemic anxiety on their employment confidence and employment situation perception. The survey shows that 74.03% of college students feel anxious about the COVID-19, of which 30.21% have serious anxiety, 25.62% are moderate anxiety, and mild anxiety accounts for 18.20%. The anxiety of college students may be related to the influence of COVID-19 on their study, life, interpersonal communication and employment (Sun et al., 2021). In addition, nearly 50% of college students felt general or no confidence in employment, and 73.32% of college students believed that the current employment situation of the COVID-19 was not optimistic or general. College students' low confidence in employment and a strong sense of crisis in the employment situation are also closely related to the heavy damage COVID-19 has brought to the global economy. The economic downturn has caused the depression of the employment market, the normal employment interview is often blocked, college students have difficulties in going abroad to study, the internal employment competition is intensifying, and there is a serious “Involution” phenomenon^4^ in China (Wang and Zhang, 2022), which leads college students to face the employment problem with a more pessimistic attitude.

Previous studies have shown that college students' anxiety during the epidemic has a certain negative impact on employment. This study also confirmed this conclusion through the results of the ordinal multiple logistic regression, and expanded and supplemented it. The results show that COVID-19 anxiety has a negative impact on Chinese college students' employment confidence and employment situation perception. In recent years, the global economic downturn, domestic economic downward pressure and COVID-19 have made the employment situation of college graduates more severe. The increasing anxiety about the epidemic has caused psychological burden on college students, making it more difficult for college students to obtain the desired jobs when looking for a job (Gao et al., 2021). Therefore, college students' employment confidence is also more vulnerable to attack, their perception of the employment situation is more pessimistic, and their sense of employment crisis is enhanced. In addition, from the statistical analysis results of the control variables, employment guidance helps college students build employment confidence and judge the employment situation with an optimistic attitude. College students who have received employment guidance will have a clearer plan for their career, a clearer employment goal when choosing a job (Ma et al., 2021), and have stronger employment confidence. At the same time, with the growth of college students' age and the improvement of their educational background, college students' psychological quality is gradually mature and their ability to work and interpersonal communication is continuously improved, their employment confidence is enhanced, and their sense of crisis in the employment situation is reduced. From a regional perspective, the overall economic level and employment situation in the eastern region are far better than those in the central and western regions (Yuan et al., 2018). College students are more likely to find a satisfactory job in the eastern region, so they have stronger confidence in employment. However, when the salary level of employment cannot meet expectations, the higher the expectation of monthly salary, it will reduce the employment confidence of college students.

From the perspective of gender and major differences, compared with female college students, male college students' epidemic anxiety can weaken their employment confidence to a greater extent and have a higher sense of crisis in the employment situation. On the one hand, this may be related to the Chinese thought of “Male in Charge of The Outside, Female in Charge of The Inside”. Male are given the responsibility of “supporting the family” in society, and there is great pressure on employment. On the other hand, male pay more attention to the money and power that work can bring , for example, male are more eager to seek higher paid jobs and male are longing for higher positions (Yang and Zhao, 2020). From these two levels, male have higher requirements for employment. The destruction of the employment environment caused by the epidemic has led to the increase of employment uncertainties and the lack of a large number of high-quality jobs (Phyllis et al., 2020), which has made male college students increasingly anxious about the epidemic and have a stronger sense of crisis in the employment situation, affecting their confidence in finding a satisfactory job. Compared with college students majoring in Liberal Arts and History, college students majoring in Science and Engineering have higher requirements for salary level. College students majoring in Science and Engineering have strong professional skills and believe that their professional value is high, such as electronic information technology, urban planning, construction engineering, chemical engineering, etc., and do not consider employment positions whose salary is lower than expected. However, affected by the epidemic, the wage level in the employment market has declined, the supply of high paying jobs has decreased, and the employment competition has intensified (Wang et al., 2022), leading to the epidemic anxiety, which has a greater negative impact on the employment confidence and employment situation perception of Science and Engineering college students.

Active employment guidance can effectively reduce college students' anxiety about the epidemic, and has a moderating effect on college students' employment confidence and employment situation perception. The opening of employment guidance work and guidance courses in colleges and universities is an important window for college students to learn workplace skills, obtain employment market information, build career planning, and form a positive outlook on employment. College students who have received employment guidance are not only trained to obtain reliable employment information, grasp the changes of employment situation and job selection skills (Mahir et al., 2021). Moreover, it can also get the help of instructors on psychological counseling, alleviate the anxiety of college students caused by the impact of the epidemic, develop a positive attitude toward employment, and be able to methodically look for employment opportunities and actively face employment difficulties in COVID-19 (Falcón-Linares et al., 2021). Compared with college students who have not received employment guidance, they have a more optimistic attitude toward employment, a sense of crisis in the employment situation has been alleviated, and their employment confidence has been enhanced. The results show that active employment guidance is very necessary for college students.

## Conclusion

74.03% of college students were anxious about the COVID-19, of which 73.32% were not optimistic about the employment situation. Nearly 50% of college students have low confidence in employment. There is a significant negative correlation between epidemic anxiety and Chinese college students' employment confidence, and there is a negative correlation between epidemic anxiety and employment situation perception, but it is not significant. The stronger the epidemic anxiety, the stronger the sense of crisis in the employment situation, and the weaker the employment confidence. Employment guidance, older age and higher education have a significant positive impact on college students' employment confidence and employment situation perception. College students in the eastern region have higher employment confidence and more optimistic perception of the employment situation, but the higher the monthly salary, the lower the employment confidence. Compared with female college students and Liberal Arts and History students, male college students and Science and Engineering students' epidemic anxiety have a stronger negative impact on employment confidence and employment situation perception. Employment guidance for college students can help alleviate the anxiety of college students, enhance their confidence in employment and reduce their sense of crisis in the employment situation. Employment guidance plays an important role in moderating the relationship between epidemic anxiety and employment confidence and employment situation perception.

College Students' epidemic anxiety has an important impact on their employment. Combined with the research results, the enlightenment to us is that the state and universities should start from the following aspects to promote the employment of college students. First, colleges and universities should pay attention to psychological counseling of college students. College student counselors, tutors and psychological counseling departments in colleges and universities should timely find and eliminate college students' anxiety or depression, encourage college students to maintain good work and rest habits, strengthen outdoor exercise, and reduce the anxiety caused by the epidemic (Li and Chen, 2022). Second, strengthen employment guidance in colleges and universities. Deliver the latest employment information, urge college students to build career plans, change their employment concepts, reasonably position employment goals, and adjust employment expectations. Pay special attention to the employment guidance for male college students and Science and Engineering students, and combine the employment guidance with mental health education, and always pay attention to the mental health problems of college students (Wang et al., 2019). Third, vigorously support the development of small, medium-sized and micro enterprises. Small, medium-sized and micro enterprises are the main force to accommodate the employment of a large number of college graduates, ensure sufficient employment positions, guide college students to work in private enterprises, and avoid excessive pursuit of employment positions in state-owned enterprises and government agencies^5^ (Zhang and Gao, 2022). Fourth, improve the employment and entrepreneurship service system for college students. Strengthen the employment assistance for fresh graduates, such as employment subsidies, employment position recommendation, etc. Build an employment and entrepreneurship service system for college students based on big data technology, dynamically monitor the employment situation of college students, and use big data to link enterprises and colleges (Li, 2022), so as to solve the employment problem of college students faster and better.

## Data availability statement

The raw data supporting the conclusions of this article will be made available by the authors, without undue reservation.

## Author contributions

SZ supervised and directed. SZ and JZ conceived and designed the experiments. GW and JZ wrote the original draft and performed data analysis and interpretation. GW reviewed and edited the manuscript. GW and WC performed data collection and collation. All authors contributed to the article and approved the submitted version.

## Funding

This study was supported by Fujian Provincial Natural Science Foundation (Grant No. 2020J01583), Annual Project of the 13th Five Year Plan of Educational Science in Fujian Province (Grant No. FJJKCG20-174), and Science and Technology Innovation Fund of Fujian Agriculture and Forestry University (Grant Nos. CXZX2019036, CXZX2017373, and CXZX2017587).

## Conflict of interest

The authors declare that the research was conducted in the absence of any commercial or financial relationships that could be construed as a potential conflict of interest.

## Publisher's note

All claims expressed in this article are solely those of the authors and do not necessarily represent those of their affiliated organizations, or those of the publisher, the editors and the reviewers. Any product that may be evaluated in this article, or claim that may be made by its manufacturer, is not guaranteed or endorsed by the publisher.

## Footnotes

^1^Source: Ministry of Education News. http://www.moe.gov.cn/fbh/live/2021/53931/mtbd/202112/t20211229_591299.html.

^2^Source: Website of the Ministry of Education. http://www.moe.gov.cn/srcsite/A15/s3265/202111/t20211119_581056.html.

^3^Kessler10 scale is a short scale that can be used to investigate the mental health status of people. It was first compiled by Kessler and Mroczek of the University of Michigan in the early 1990s. The scale contains 10 items, which are the frequency of nonspecific mental health-related symptoms such as anxiety and stress levels experienced in the past 4 weeks. Kessler10 scale has good reliability and validity, and is suitable for large-scale mental health surveys.

^4^The original meaning of the word “Involution” refers to the phenomenon that human society stagnates or cannot be transformed into another advanced mode after reaching a certain form in a development stage. In short, it means “growth without development”, “ineffective efforts”, “excessive competition”, etc. During the epidemic period, after spreading on the Internet, many college students used it to refer to irrational internal competition or “forced voluntary” competition. Now it also refers to the phenomenon that the same industry competes to pay more efforts to compete for limited resources, resulting in the decline of individual “income effort ratio”. It can be seen as “hard inflation”.

^5^The survey shows that 70.84% of college students intend to work in party and government organs, state-owned institutions and state-owned enterprises, while the proportion of college students who intend to work in collective enterprises, private enterprises, foreign-funded enterprises and other types of enterprises is only 7.16, 8.04, 6.18, and 7.77%, respectively.
